# Inter-correlation of knowledge, attitude, and osteoporosis preventive behaviors in women around the age of peak bone mass

**DOI:** 10.1186/1472-6874-14-35

**Published:** 2014-03-03

**Authors:** Ploynin Puttapitakpong, Sukanya Chaikittisilpa, Krasean Panyakhamlerd, Chaichana Nimnuan, Unnop Jaisamrarn, Nimit Taechakraichana

**Affiliations:** 1 Menopause Research Unit, Department of Obstetrics and Gynecology, Faculty of Medicine, Chulalongkorn University, Bangkok 10330, Thailand; 2 Department of Psychiatry, Faculty of Medicine, Chulalongkorn University, Bangkok 10330, Thailand

**Keywords:** Knowledge, Attitude, Behavior, Osteoporosis

## Abstract

**Background:**

As silent and preventable in nature, postmenopausal osteoporosis awareness should be raised among young women prior to an irreversible period of declining bone mass. We therefore decided to assess the inter-correlation of knowledge, attitude and osteoporosis preventive behaviors in women around the age of peak bone mass.

**Methods:**

A cross-sectional study was conducted in 430 women aged 20–35 years. The participants’ knowledge, attitude and behaviors concerning osteoporosis prevention were assessed along with demographic data using a four-part questionnaire. The items in this questionnaire were established by extensive literature review, including the Guideline for Management of Osteoporosis of the Thai Osteoporosis Foundation (TOPF) 2010. The content was validated by experts in osteoporosis and reliability was obtained with a Cronbach’s alpha score of 0.83.

**Results:**

The mean age of women in this study was 29.4 ± 4.6 years. Half of the participants (49.5%) had heard about osteoporosis, mostly from television (95.3%, n = 203/213) and the internet (72.8%, n = 155/213). Most women had certain knowledge (85.2%) and positive attitude towards osteoporosis (53.3%). Nevertheless, 80% of the studied population did not have appropriate osteoporosis behaviors. We found significant correlation between the level of attitudes and osteoporosis behaviors (adjusted odd ratio = 3.3 with 95% confidence interval of 1.9-5.7); attitude and educational level (adjusted odd ratio = 2.2 with 95% confidence interval of 1.4-3.4); and attitude and knowledge (adjusted odd ratio = 3.5 with 95% confidence interval of 1.8-6.8).

**Conclusion:**

Despite having certain knowledge about osteoporosis, the young women did not seem to have appropriate osteoporosis preventive behaviors. Developing a right attitude towards osteoporosis may be a key determinant to improving health practices in order to prevent osteoporosis.

## Background

Osteoporosis is a skeletal disease characterized by low bone mass and microarchitectural deterioration of bone tissue, leading to bone fragility and increased risk of fractures [1]. The World Health Organization (WHO) has defined osteoporosis in terms of bone mineral density which is more than 2.5 SD below the peak young adult mean. Based on these criteria, it has been estimated that 13–18% of women aged 50 and over have osteoporosis [2]. For those over the age of 80, the proportion rises to 70% [3].

Osteoporosis is often referred to as “silent disease”. The balance of bone turnover shifts in favor of bone resorption through adulthood until late in life [4,5]. With a striking increase in aging population, the number of elderly people with osteoporosis-related fractures increases substantially. The pain, suffering and economic costs will be enormous [6]. Despite its adverse effects, osteoporosis is often overlooked and undertreated. About 75% of all women aged 45–75 years have never discussed about osteoporosis with their physician. In difference to identified patients at-risk, the lack of interest in educating the public, and nonchalance to implementation of preventive measures may lead to tragic consequences [7].

As a preventable disease, it is cost-effective to encourage osteoporosis preventive behavior, for example, adequate calcium intake, optimal exposure to sunlight to induce vitamin D production in skin, regular weight-bearing exercise, smoking cessation and avoidance of excessive caffeine drinking [8,9]. Peak bone mass enhancement in young age, bone loss reduction after menopause, and fall preventive measures are important to minimize fracture risk. The challenge for osteoporosis prevention programs is to identify young populations at risk and encourage the adoption of risk-reduction behaviors. Prior to raising disease awareness, we need to have baseline information concerning general knowledge and osteoporosis preventive behavior of our population. An understanding of the attitudes of young women on osteoporosis is essential for the development and delivery of an effective health promotion program [2].

Several studies revealed that women had poor knowledge of osteoporosis, which may result in inadequate preventive health behaviors [10,11]. Strikingly, most of the available Knowledge-Attitude-Practice studies on osteoporosis were conducted in postmenopausal women whose bone losses had already accelerated [12-14]. In fact, primary prevention of the disease should be started at younger ages in order to maximize peak bone mass.

## Methods

We conducted a cross sectional survey among women attending the Gynecology Clinic, King Chulalongkorn Memorial Hospital between July and October 2010. This study was approved by the Institutional Review Board, Faculty of Medicine, Chulalongkorn University.

A total of 430 women aged 20 to 35 years who never had formal health training on osteoporosis were enrolled. All of them agreed to participate in the present study and oral informed consent were given. They were advised to complete a standardized questionnaire. The questionnaire consisted of four parts. The first part included background information, i.e. demographic, social and educational data, economic status, as well as asking whether they had ever heard about osteoporosis. The second part included 11 questions on osteoporosis preventive behaviors, e.g. intake of food containing calcium, practice of weight bearing exercise and exposure to sunlight. The third part included 18 questions on attitude toward osteoporosis. These questions were scored in four scales, from “4” as “completely agree” to “1” as “completely disagree”. The last part was the participants’ knowledge about osteoporosis. There were 23 questions about osteoporosis and its risk factors. A total score for knowledge was obtained from point-summation of all questions. Only those who made a correct answer would get onepoint for each particular question.

The content of the questionnaire was developed by an extensive literature review [12-16] including the Guideline for Management of Osteoporosis of the Thai Osteoporosis Foundation (TOPF) 2010 by three experienced experts in osteoporosis, to ensure that its content was correct and clear. The questions that were included were based on the theoretical assumptions of social cognitive models in general and the Health Belief Model in particular.

We also conducted a pilot study in a cohort of 25 women from the Gynecology Clinic, King Chulalongkorn Memorial Hospital to ensure reliability and clarity of the questionnaire. Cronbach’s alpha coefficient of the attitude scales was 0.83.

### Statistical analysis

Analyses were conducted using SPSS version 17.0. Descriptive statistics (means, SDs, percentages) were used to describe the population characteristics of the study population and main variables: level of knowledge, attitude and osteoporosis preventive behavior. Chi-squared test was performed onknowledge, attitude, economic and educational status to reveal factors influencing osteoporosis preventive behaviors. Correlation analysis was used to determine the correlation between knowledge and osteoporosis preventive behavior and between attitude and osteoporosis preventive behavior. A probability value (P-value) of less than 0.05 was accepted as a trend to be statistically significant. Binary logistic regression analysis was carried out for the correlation between educational level, economic status, knowledge, attitude about osteoporosis and osteoporosis preventive behavior.

## Results

A total of 430 women completed the questionnaire. The mean age was 29.4 ± 4.6 years, and age range was 20 to 35 years old of which 54.4% of participants was married. Most of the women (63.5%) had an average income of less than 15,000 baht/month. About half of the studied population (54%) had a high level of education (Table 1).

**Table 1:** Population characteristics (n = 430)

***Variables***	***n (%)***
***BMI (kg/m***^***2***^ ***)***	
- < 18.0	59 (13.7)
- 18.0 – 25.0	293 (68.1)
- > 25.0	75 (17.4)
***Marital status***	
- Single/divorced	191 (44.4)
- Married	234 (54.4)
- Others	4 (0.9)
***Education***	
- Bachelor’s degree and above	232 (54.0)
- Below bachelor’s degree	198 (46.0)
***Occupation***	
- Employee	148 (34.3)
- Government officer	80 (18.6)
- Merchant	56 (13.0)
- Entrepreneur	48 (11.1)
- Housewife	47 (10.9)
- Others	48 (11.1)
***Family’s income (Baht/Month)***	
- < 15,000	273 (63.5)
- ≥ 15,000	150 (34.9)

About 49.5% of participants had heard about osteoporosis, the information is mostly from television and internet (Table 2).

**Table 2:** Source of information (n = 203)

**Source**	**n (%)**
Television	203 (95.3)
Internet	155 (72.8)
Newspaper	83 (39.0)
Friends	77 (36.2)
Doctor/nurse	65 (30.5)
Others	34 (16.0)

The mean score of knowledge about osteoporosis was 18.5 ± 3.0 points from a possible 23. Most participants had adequate knowledge about osteoporosis (85.2%) and 53.3% of women had a good attitude towards the prevention of osteoporosis. However, 80% of women were found to have inappropriate osteoporosis preventive behavior.

When using Chi-squared to test the relationship among the level of knowledge, attitude, education level and economic status with osteoporosis preventive behaviors we found significant correlation between osteoporosis preventive behavior with attitude (p < 0.001) and educational status (p < 0.01). Nevertheless, there was no statistical significance in the relationship between economic status (p = 0.39) and level of knowledge (0.08) with osteoporosis preventive behaviors.

When analyzing the data with binary logistic regression, we found statistically significant correlation only between the level of attitude toward osteoporosis and osteoporosis preventive behavior (adjusted odd ratio = 3.3, 1.9-5.7 95% confidence interval). We also found significant correlation between attitude toward osteoporosis and educational level (adjusted odd ratio = 2.2, 1.4-3.4 95% confidence interval) and knowledge (adjusted odd ratio = 3.5, 1.8-6.8 95% confidence interval).

## Discussion

This study evaluated the knowledge, attitude and osteoporosis preventive behaviors of young Thai women who attended the Gynecology Clinic, King Chulalongkorn Memorial Hospital. Two hundred and thirteen women (49.5%) in our survey had heard about osteoporosis, mostly through television (95.3%) and internet (72.8%). Only thirty percent of them obtained the information from a doctor, nurse or midwife. Our data suggest that health care providers have either lost opportunities to disseminate osteoporosis information to young women or such information has not been appropriately received and well retained. It is possible that physicians have traditionally been trained to practice curative medicine. They may not place sufficient emphasis on preventive care. At present, young women are interested in getting information from online social networking and from television. In order to provide knowledge about osteoporosis to this age group, it is better to convey it through appropriated media that is suitable with their lifestyle and interests.

Most of the women in this study had adequate knowledge about osteoporosis (85.2%) when compared to prior studies in postmenopausal Thai women [11-13]. This is probably due to the overall improvement of education level and better health information about osteoporosis. Although the study revealed a high proportion of women with adequate knowledge and a good attitude toward osteoporosis, the number of women with inappropriate osteoporosis preventive behavior in this young age group was high. This finding reflects that despite having certain knowledge about osteoporosis, young women still lack adequate insight into the consequences of the disease, insight which might result in proper osteoporosis preventive behaviors. This is inconsistent with the findings of other reports [17] that women in our study may not have been sufficiently motivated to engage in preventive behaviors.

Our study revealed significant association between attitude toward osteoporosis and osteoporosis prevention behaviors while there was no significant correlation between knowledge and osteoporosis preventive behaviors.This finding showed that good attitude was very important to osteoporosis prevention behaviors.

Further analysis showed a significant correlation between educational levels, knowledge and attitude toward osteoporosis. The women with higher education and adequate knowledge were more likely to have a good attitude toward osteoporosis. This result may imply that higher education may open learning horizons, providing access to knowledge, which results in good attitude.

## Conclusion

This study suggests that general knowledge about osteoporosis that young women learn mostly from television and internet may not be adequate to change their attitudes toward osteoporosis, which could result in changing of osteoporosis preventive behaviors. Women in this age group are in their critical period to enhance peak bone mass. Therefore, it is necessary to find effective education programs that have an influence over attitude and health preventive behaviors, as osteoporosis and its fracture consequences are preventable conditions.

## Competing interests

The authors declare that they have no conflicts of interest.

## Authors’ contributions

PP: is the principle investigator who did all the work from conception, design and research conduct til manuscript development. SC: is the principle co-investigator who is the gynecologist expert in the field of quality of life with contributions to conception and design, questionnaire development, acquisition of data, analysis, interpretation of data and manuscript development. KP: is a co-investigator who is the gynecologist expert in the field of osteoporosis who helped with conception, design, analysis, interpretation and giving final approval of the version to be published.CN: is a co-investigator who is the psychiatrist expert in the field of quality of life with contributions to conception and design, questionnaire development and standardization, analysis, interpretation of data and giving final approval of the version to be published. UJ: is a co-investigator who is the gynecologist and ethical expert who helped with conception, design, analysis, interpretation and giving final approval of the version to be published. NT: is a co-investigator who is the gynecologist expert in the field of osteoporosis who helped with conception, design, analysis, interpretation, critical manuscript revision and giving final approval of the version to be published. All authors read and approved the final manuscript.

## Pre-publication history

The pre-publication history for this paper can be accessed here:

http://www.biomedcentral.com/1472-6874/14/35/prepub
